# Relationship Between Psychological Distress and Cognitive Function Differs as a Function of Obesity Status in Inpatient Heart Failure

**DOI:** 10.3389/fpsyg.2020.00162

**Published:** 2020-02-14

**Authors:** Alice V. Ely, Courtney Alio, Desiree Bygrave, Marykate Burke, Earl Walker

**Affiliations:** ^1^Department of Psychiatry, Christiana Care, Newark, DE, United States; ^2^Department of Psychiatry, University of Pennsylvania, Philadelphia, PA, United States; ^3^School of Nursing, University of Delaware, Newark, DE, United States

**Keywords:** heart failure, obesity, cognition, depression, anxiety

## Abstract

Heart failure (HF) is a chronic medical condition rapidly growing in prevalence. Evidence links HF to cognitive decline, obesity, and psychological distress. The current study examined the association between cognitive function and ejection fraction (EF%), anxiety, depression, and obesity in inpatient HF. Patients completed the Generalized Anxiety Disorder 7-Item Scale (GAD-7), Patient Health Questionnaire 9-Item Scale (PHQ-9), and Mini-Cog while hospitalized for HF. Additional demographic and medical information was gathered via chart review. All models controlled for age. Of 117 patients assessed (49% male), 55% (*n* = 64) were obese. ANCOVA analyses were conducted comparing those with obesity and without on cognitive function: model A included EF%, model B included depression, and model C included anxiety. All three models were significantly related to cognitive function. There was a significant interaction effect of EF% and obesity and of anxiety and obesity to predict Mini-Cog scores. *Post hoc* partial correlational analyses revealed that anxiety was negatively associated with Mini-Cog scores among only patients without obesity. Depression was not significantly related to cognitive function in either group. However, patients with obesity demonstrated higher depression and anxiety than patients without. Results suggest that at lower EF%, and with higher anxiety, patients without obesity may be at greater risk of cognitive dysfunction than those with obesity. Cognitive dysfunction among HF patients with obesity may be independent of psychological distress. These findings may reflect the “obesity paradox” observed among HF patients, in that patients with obesity may have a different biopsychosocial presentation, which may lead to unexpected clinical outcomes. Further research is necessary to articulate the relationship of obesity and cognitive function in HF.

## Introduction

Heart failure (HF) is rapidly growing in prevalence, affecting over 5 million U.S. adults, with several hundred thousand more diagnosed each year (Mozaffarian et al., 2016; Ziaeian and Fonarow, 2016). HF accounts for 5% of all hospitalizations, with up to a quarter of those patients readmitted within 30 days (Krumholz et al., 2009; Bergethon et al., 2016). These hospitalizations result in significant economic and public health burden (Ziaeian and Fonarow, 2016). Given the chronicity of HF, it is critically important to identify risk factors for repeated hospitalizations.

Growing evidence links HF with a disease-specific (Leto and Feola, 2014), progressive decline in cognitive function (Van Den Hurk et al., 2011; Almeida et al., 2012; Hjelm et al., 2012), with nearly 3/4 of HF patients showing signs of possible cognitive impairment (Hoth et al., 2008). Increased severity of HF is associated with greater cognitive dysfunction (Hoth et al., 2008; Pressler et al., 2010), although there is some evidence to the contrary (Feola, 2013). Cognitive dysfunction has been associated with poor self-care (Leto and Feola, 2014) and low adherence to medication and treatment recommendations (Alosco et al., 2012b; Hawkins et al., 2012; Agarwal et al., 2016; Huynh et al., 2016; Dolansky et al., 2017), potentially contributing to increased HF morbidity, hospitalization, and death (Agarwal et al., 2016; Huynh et al., 2016). Given the established impact of cognitive dysfunction on medical outcomes in HF, it is of particular interest to examine factors that interact to predict cognitive dysfunction in this population to better understand variables that increase risk for frequent hospitalization.

Elevated body mass index (BMI, weight in kg/height in m^2^) is independently related to reduced performance on measures of learning, memory, executive function, and global cognition in otherwise healthy individuals (Stanek et al., 2011; Dye et al., 2017). More than 40% of HF patients are obese (Kapoor and Heidenreich, 2010) and obesity may exacerbate cognitive deficits in the HF population (Alosco et al., 2012a, 2014c). However, the way in which elevated BMI interacts with HF severity to promote cognitive dysfunction is unclear.

In healthy individuals, anxiety and depression are also independently associated with poorer cognitive function (McDermott and Ebmeier, 2009; Rock et al., 2014; Snyder et al., 2015; Shields et al., 2016). Psychological distress is quite prevalent in HF, with depression affecting up to 60% of patients (Yohannes et al., 2010) and up to 55% of patients demonstrating elevated anxiety (Easton et al., 2016). Both depression and anxiety in HF are risk factors for reduced quality of life, poor self-care, and higher rates of hospitalization and mortality (Yohannes et al., 2010; Sherwood et al., 2011; Kato et al., 2012; Ketterer et al., 2014; Suzuki et al., 2014; Alhurani et al., 2015; Tovar et al., 2016). While a growing literature demonstrates a relationship between depression and cognitive dysfunction in cardiovascular disease in general (Armstrong et al., 2018), and HF in particular (Foster et al., 2011; Garcia et al., 2011; Alosco et al., 2014a; Hawkins et al., 2015a), it is unknown how anxiety impacts cognitive function in HF. A better understanding of mechanisms through which depression and anxiety may interact with HF severity to exacerbate cognitive dysfunction is an important step toward improving self-care and outcomes for this patient population.

It is essential to identify risk factors for poor self-care and repeated hospitalizations among HF patients. By investigating relationships among obesity, depression, anxiety, EF%, and cognitive function in HF, we can identify potential targets for cognitive and behavioral health treatment and contribute to improved quality of life for this population. While evidence suggests that obesity, psychological distress, and HF severity are independently related to cognitive dysfunction in HF, the current study aimed to address gaps in the literature by examining the interactive relationship between these variables. It was hypothesized that obesity and psychological distress would be related to reduced cognitive function, potentially interacting with HF severity.

## Materials and Methods

### Participants

Assessment was completed as a part of a quality improvement project at Christiana Hospital in Newark, DE, United States. Participants were current inpatients who were identified as being at high risk for 30 day re-admission to the hospital using model-driven machine learning technology described in greater detail in the Supplementary Material. Upon identification, they were assessed for criteria that would limit their ability to attend outpatient appointments with the Advanced Heart Failure service at Christiana, and for medical comorbidities that would take precedent at that hospital visit, such as end-stage renal failure or severe lung disease. They were then referred for assessment by the Heart Failure Task Force multidisciplinary team. As part of this pilot program to incorporate cognitive and behavioral health screening into inpatient HF treatment, 117 patients were assessed as a part of routine clinical care. Of those patients, 49% were male, and the mean age was 72.6 years (*SD* = 11.46). Patients with obesity (BMI ≥ 30 kg/m^2^) made up 55% (*n* = 64) of the sample. Patients had both preserved and reduced ejection fraction (EF%), a measure of the amount of blood pumped from the left ventricle with each heartbeat and one indicator of HF severity and risk (Cikes and Solomon, 2016). Analyses were conducted upon completion of 6 months of the quality improvement project.

These *post hoc* analyses were determined to be exempt from 45 CFR 46 Research Regulations by the Institutional Review Board of Christiana Care. A Waiver of HIPAA Authorization was granted, given the study involved no more than minimal risk to the privacy of individuals and as such consent was not required.

### Inclusion Criteria

To be included in analyses, patients must (1) have had a current diagnosis of HF or cardiomyopathy as determined by inpatient cardiologists trained in advanced HF diagnosis and treatment, (2) be between the ages of 18 and 100 years old, (3) be proficient in written and spoken English, (4) be willing and able to write, and (5) have at least an 8th grade education.

### Exclusion Criteria

Patients were excluded from analyses if they (1) had a diagnosis of neurological or seizure disorder, (2) met diagnosis of alcohol or drug dependence in the 3 months prior to assessment, (3) had a diagnosis of a severe major affective or anxiety disorder or presence of other psychopathology that might interfere with ability to participate in the study (e.g. requiring inpatient hospitalization), (4) were diagnosed with organic brain syndromes, dementia, psychotic disorders or severe intellectual disability, (5) demonstrated delirium or altered mental status on assessment days.

### Measures

#### Mini-Cog (Borson et al., 2000)

The Mini-Cog consists of a three-item memory/recall test and a clock-drawing test to measure cognition. The Mini-Cog is scored on a 5-point scale, with lower scores indicating greater cognitive dysfunction. A cut point of <4 may indicate cognitive impairment and need for further evaluation of cognitive status. It is a reliable, valid measure for assessing clinically significant cognitive impairment (Borson et al., 2003, 2005; Patel et al., 2015), though Cronbach’s alpha has been found to be low (0.278) (Costa et al., 2012), likely due to it comprising only two questions. The Mini-Cog has been used in prior studies of HF and has been shown to predict posthospitalization readmission risk (Patel et al., 2015; Agarwal et al., 2016).

#### Patient Health Questionnaire (PHQ-9; Kroenke et al., 2001)

Depressive symptoms were measured with this 9-item, self-report measure reflecting the diagnostic criteria for major depressive disorder. Patients were asked to rate how often each symptom has bothered them during the past 2 weeks on a rating scale from 0 (not at all) to 3 (nearly every day). Total scores range from 0 to 27, with higher scores indicating a greater severity of depression. It is a reliable, valid measure of depressive symptoms in patients with HF (Cronbach’s α = 0.85) (Hammash et al., 2013).

#### Generalized Anxiety Disorder 7-Item Scale (GAD-7; Spitzer et al., 2006)

Anxiety symptoms were measured with this 7-item self-report measure reflecting the diagnostic criteria for generalized anxiety disorder. Patients were asked to rate how often they have been bothered by the described symptoms over the last 2 weeks using a 4-point rating scale from 0 (not at all) to 3 (every day). Total scores range from 0 to 21, with higher scores reflecting higher severity levels of generalized anxiety disorder symptomology. It has good reliability (Cronbach’s α = 0.89), as well as criterion, construct, factorial, and procedural validity (Löwe et al., 2008).

### Procedure

Assessors were masters- and doctoral-level members of the cardiovascular behavioral health (CVBH) clinical team trained in administering measures. CVBH assessors administered the Mini-Cog, PHQ-9, and GAD-7 with patients during their inpatient hospitalization in their hospital room. Following assessment, CVBH provided feedback, psychoeducation, and referrals if necessary to all patients. Assessments were completed within a week of admission to the Advanced HF service. Patients were typically discharged within 5 days following assessment. Further data regarding demographic and medical information, including BMI and EF%, was collected via chart review following initial evaluation. All patients were scheduled to attend outpatient appointments in the HF clinic following hospital discharge for continuity of care.

### Statistical Analyses

All statistical analyses were performed using IBM SPSS Statistics for Windows, Version 24.0. In order to characterize the relationship of cognitive function with EF%, psychological distress, and obesity status, three ANCOVA analyses were conducted, adjusting for age and sex. Two *post hoc* partial correlational analyses were conducted within each group (those with and without obesity) to determine if the relationships between cognitive function and other variables differed based on obesity status. *Post hoc* power analysis using G^∗^Power (Faul et al., 2007) determined the sample size of 118 was adequately powered (1-β = 0.82) with the achieved effect size of the corrected ANCOVA models (f2 = 0.33; α = 0.05). Power analysis suggests the sample size is too small to adequately power an interaction effect, and as such these results should be interpreted with caution.

## Results

### Descriptive Data (Table 1)

Male and female participants did not significantly differ on age, nor on cognitive function, depression, anxiety, or BMI with or without controlling for age. Men (*m* = 35.26, *SD* = 15.77) had a significantly lower EF% (*F* = 6.05, *p* = 0.015) than women (*m* = 42.98, *SD* = 17.7). Participant BMI ranged from 19.8–59.8 kg/m^2^. Patients with obesity were significantly younger than those without (both with and without controlling for sex), and had significantly lower EF% when controlling for age and sex. Patients with obesity did not differ significantly on Mini-Cog scores but did report significantly greater depression (*p* = 0.01) and anxiety (*p* = 0.019) compared to those without, adjusting for age and sex.

**TABLE 1:** Descriptive data for the full sample.

	*N*	Mean	*SD*	Minimum	Maximum
Age (years)	117	72.6	11.5	38	94
BMI (kg/m^2^)	117	32.8	8.7	19.8	59.8
EF (%)	114	39.5	17.2	10	75
Mini-Cog	116	3.1	1.5	0	5
PHQ9	116	6.4	4.6	0	21
GAD7	116	4.3	5.0	0	20

### Obesity and Ejection Fraction (Table 2A)

Controlling for age and sex, the model of obesity, EF%, and their interaction was significantly associated with Mini-Cog score (*F* = 3.38, *p* = 0.017, η2 = 0.12). There was a significant interaction effect of EF% and obesity (*F* = 4.06, *p* = 0.046, η2 = 0.04) such that lower EF% was related to worse performance in patients without obesity, but better performance in those with obesity. The main effect of obesity was related to Mini-Cog score at a trend level, while the main effect of EF% was non-significant.

**TABLE 2A:** Relationship between obesity, ejection fraction (EF%) and cognitive function as measured by Mini-Cog score, controlling for age.

	df	Mean square	*F*	*p*	η2
Corrected Model	5	6.03	2.9	0.017	0.12
Intercept	1	74.9	35.8	<0.001	0.25
Age	1	17.8	8.5	0.004	0.07
Sex	1	1.5	0.7	0.398	0.01
Obesity	1	6.7	3.2	0.077	0.03
EF%	1	1.7	0.8	0.364	0.01
Obesity * EF%	1	8.5	4.1	0.046	0.04
R Squared = 0.119 (Adjusted R Squared = 0.078)					

### Obesity and Depression (Table 2B)

Controlling for age and sex, the model of obesity, PHQ-9 score, and their interaction was significantly associated with Mini-Cog score (*F* = 2.59, *p* = 0.03, η2 = 0.12). The interaction of depression and obesity was not significantly related to cognitive function (*F* = 0.28, *p* = 0.6). The main effect of depression was related to Mini-Cog score (*F* = 4.12, *p* = 0.045, η2 = 0.036), with higher depression linked to lower cognitive function, while the main effect of obesity was non-significant. Adding EF% as a covariate did not substantially change the strength of the model (*F* = 2.25, *p* = 0.044, η2 = 0.11).

**TABLE 2B:** Relationship between obesity, depression (as measured by the Patient Health Questionnaire, PHQ-9) and cognitive function (as measured by Mini-Cog score), controlling for age and ejection fraction (EF%).

	df	Mean square	*F*	*p*	η2
Corrected Model	6	4.8	2.3	0.044	0.11
Intercept	1	78.3	36.6	<0.001	0.26
Age	1	16.6	7.8	0.006	0.07
Sex	1	1.7	0.8	0.382	0.01
EF	1	1.0	0.5	0.495	0.00
Obesity	1	0.4	0.2	0.664	0.00
PHQ9	1	6.4	3.0	0.086	0.03
Obesity * PHQ9	1	1.3	0.6	0.435	0.01
R Squared = 0.114 (Adjusted R Squared = 0.063)					

### Obesity and Anxiety (Table 2C)

Controlling for age and sex, the model of obesity, GAD-7 score, and their interaction was significantly associated with Mini-Cog score (*F* = 3.41, *p* = 0.007, η2 = 0.14). The interaction of obesity with anxiety was significantly linked to Mini-Cog score (*F* = 4.03, *p* = 0.047, η2 = 0.036). The main effect of anxiety was related to Mini-Cog score (*F* = 7.49, *p* = 0.007, η2 = 0.064), with higher anxiety linked to lower cognitive function, while the main effect of obesity was non-significant. Adding EF% as a covariate did not substantially change the strength of the model (*F* = 3.15, *p* = 0.007, η2 = 0.15).

**TABLE 2C:** Relationship between obesity, anxiety (as measured by the Generalized Anxiety Disorder 7-item scale, GAD-7)and cognitive function (as measured by Mini-Cog score), controlling for age and ejection fraction (EF%).

	df	Mean square	F	*p*	η2
Corrected Model	6	6.3	3.1	0.007	0.15
Intercept	1	78.4	39.0	<0.001	0.27
Age	1	17.3	8.6	0.004	0.08
Sex	1	1.5	0.7	0.397	0.01
EF	1	1.8	0.9	0.348	0.01
Obesity	1	1.2	0.6	0.437	0.01
GAD7	1	15.4	7.6	0.007	0.07
Obesity * GAD7	1	7.4	3.7	0.057	0.03
R Squared = 0.152 (Adjusted R Squared = 0.104)					

### Correlations

**FIGURE 1:**
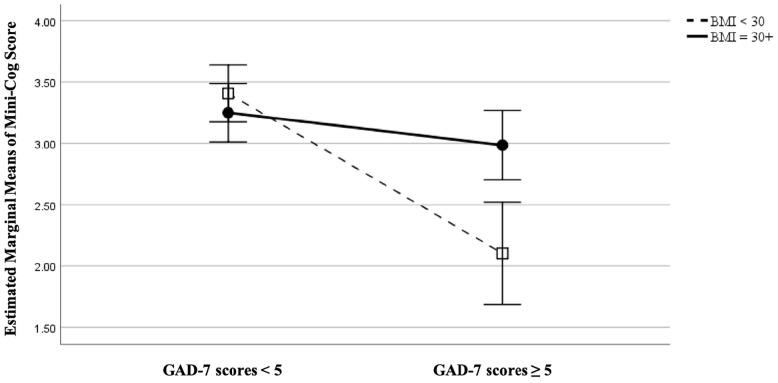
Comparing patients with obesity (BMI ≥ 30 m/kg^2^) versus those without (BMI < 30 m/kg^2^) on relationship between anxiety (as measured by the Generalized Anxiety Disorder 7-item scale, GAD-7) and cognitive function (as measured by Mini-Cog score), controlling for age at 72.6 years, EF at 39.3%, and sex.

Among patients with obesity, EF%, depression, and anxiety were not significantly related to cognitive function when controlling for sex and age. However, GAD-7 scores were negatively associated with Mini-Cog scores (*r* = −0.33, *p* = 0.018) among patients without obesity (Figure 1). Depression and EF% were not significantly related to cognitive function in patients without obesity. Notably, among those with obesity, anxiety was significantly correlated with EF% when controlling for sex and age (*r* = 0.31, *p* = 0.021). This effect was not evident in patients without obesity. Anxiety and depression were correlated significantly among both groups (*p* < 0.001).

## Discussion

We aimed to determine if interactive relationships between obesity, psychological distress, and HF severity would contribute to cognitive function, given the importance of these variables for quality of life for this population. Obesity status appears to interact with HF severity, depression, and anxiety to predict cognitive dysfunction, particularly among patients without obesity. In contrast, cognitive function in those with obesity may not vary to the same extent as a function of HF severity or psychological distress. Patients without obesity demonstrate an association between cognitive function and both EF% and anxiety, such that lower EF% and higher anxiety in this population may confer greater risk of cognitive dysfunction. These relationships are not evident in patients with obesity. However, HF patients with obesity do report higher levels of depression and anxiety than those without, and anxiety was higher among those with higher EF%. These results suggest that obesity status is linked with differing profiles of psychological distress and cognitive function, emphasizing the utility of behavioral health intervention with the inpatient HF population.

Greater psychological distress among patients with obesity is consistent with prior studies showing that BMI in general, and obesity in particular, are related to elevated rates of depression in HF (Hawkins et al., 2015b). Depressive symptoms appear to interact with obesity to impact quality of life in HF patients (Evangelista and Miller, 2006) and in cardiovascular disease more broadly (Oreopoulos et al., 2010). There is less consistent research demonstrating a relationship between anxiety and obesity in cardiovascular disease (Labad et al., 2010), but among non-clinical samples there is moderate evidence of a link between anxiety and obesity (Gariepy et al., 2010). Obesity may be associated with psychological distress in a number of ways. Weight-based discrimination and stigma can contribute to depression and anxiety (Papadopoulos and Brennan, 2015) through reduced quality of life and the awareness of others’ judgment and biases, and this distress can be compounded when experiencing chronic medical conditions. Reduced social and physical activity in obesity has been linked to higher depression and anxiety in non-clinical samples (De Wit et al., 2010), as well as in those at risk for cardiovascular disease (Bonnet et al., 2005). Furthermore, individuals with obesity demonstrate elevated HF symptoms (Clark et al., 2014), which can promote distress, and compound the physical and mobility limitations that may accompany more severe obesity.

We had predicted that obesity would predict cognitive dysfunction given prior research showing an inverse association between BMI and cognition (i.e. worsening performance at higher weights). Our results do not demonstrate this relationship. There are several possibilities that may explain this discrepancy. Alosco et al. (2012a) demonstrated a relationship between BMI and cognitive function in a more comprehensive battery of neuropsychological tests, which may have allowed for greater sensitivity than the brief Mini-Cog (Borson et al., 2000) screen. Further, we assessed patients during hospitalization, which may introduce additional variables that could influence cognitive function such as sleep (Blackwell et al., 2006), medication changes, or the mere fact that the patient’s illness is particularly exacerbated (Kindermann et al., 2012). Decompensation may have a greater impact on cognition than obesity itself, but may interact with depression and anxiety.

Patients without obesity demonstrate an association of cognitive dysfunction with higher anxiety. While anxiety has been shown to interfere with cognitive function in healthy individuals (Snyder et al., 2015; Shields et al., 2016), it is also true that cognitive dysfunction is significantly anxiety-provoking, with elevated rates of anxiety reported in treatment-seeking patients with mild cognitive impairment (Chen et al., 2018). While anxiety does not appear to predict progression to dementia (Gulpers et al., 2016), the relationship of anxiety to morbidity and mortality is somewhat unclear (Celano et al., 2018). Anxiety symptoms do not appear to be related to mortality in the HF population (Pelle et al., 2010), but in other cardiovascular populations it is associated with adverse medical outcomes (Roest et al., 2010, 2012; Celano et al., 2015). Anxiety may also independently increase risk of incident HF in patients free of cardiovascular disease (Garfield et al., 2014). Notably, this relationship was not evident in those with obesity. Given the finding that higher EF% is related to higher anxiety in these patients, and research suggesting that HF patients with obesity tend to be younger (including in this sample) (Lavie et al., 2016), hospitalization among those with better left ventricle function may be more unexpected and thus more anxiety provoking. Alternatively, patients with obesity who have a diagnosis of HF with preserved ejection fraction (HFpEF) may be more at risk for anxiety than their lean counterparts or those with reduced EF% diagnoses. More research is necessary to elucidate the relationship between EF%, anxiety, and obesity in the HF population.

The interactive relationship between psychological distress and cognitive function is also in line with findings in the biological literature. There are a number of pathways through which this association may develop (Sohani and Samaan, 2012). HF is a cardiac condition in which the heart functions below metabolic requirements (Brunwald, 2005) and as such, reductions in cognitive function may potentially be attributable to cerebral hypoperfusion and ischemia (Alosco et al., 2012a, 2013, 2015b). Both depressive symptoms and anxiety have been shown to interact with cerebral hypoperfusion to predict cognitive dysfunction in HF as well (Alosco et al., 2013, 2015a, 2014b). It is possible that HF- induced reductions in blood flow to the brain may potentiate psychological distress and cognitive dysfunction, which may in kind worsen neurobiological function. However, obesity is also linked with reduced cerebral blood flow and vascular abnormalities (Volkow et al., 2009; Willeumier et al., 2011), and while our findings do suggest elevations in depression and anxiety, these variables do not appear to predict cognitive function in patients with obesity.

Other brain-based changes in HF may play a role in this unexpected result. HF, cognitive impairment, and depression are all associated with elevated levels of proinflammatory cytokines (Pasic et al., 2003). Elevated cytokines are related to decreased levels of serotonin, which may result in depression and cognitive dysfunction (Sohani and Samaan, 2012). While it appears anxiety is related to an elevated inflammatory response in healthy individuals, there is less evidence of cytokines playing a role (Costello et al., 2019). However, higher anxiety is associated with inflammatory markers in patients with diabetes (Brennan et al., 2009), and inflammation during acute coronary syndrome has been shown to predict anxiety and cognitive symptoms of depression (Steptoe et al., 2013). Pasic et al. (2003) have argued that the increased mortality seen with depression in HF is potentially due to elevations in inflammatory markers, causing left-ventricular dysfunction. While obesity has also been shown to be associated with elevations in inflammatory biomarkers (Choi et al., 2013), patients with obesity also demonstrate higher levels of antiinflammatory adipokines, which may be protective of poor clinical outcomes (Clark et al., 2014). Elevations in adipokines are linked to depression and anxiety (Brennan et al., 2009; Bove et al., 2013; Carvalho et al., 2014), but also to higher performance on cognitive assessments (Diano et al., 2006; Lee, 2011), though there is some conflicting research (Spitznagel et al., 2010). This raises the possibility that inflammation due to obesity may contribute to psychological disturbance, but be protective of the effects of that disturbance on cognitive performance. Unfortunately we are unable to test this hypothesis with the current sample, but future research would be vital to identify the neurobiological mediators of the relationship between obesity, psychological distress, and cognitive function.

Our findings demonstrate a link between anxiety and cognition in patients without obesity, but this relationship was not evident in patients with obesity. Distress and cognitive function may be somewhat independent of each other in this obese HF sample. Previous research suggests that patients with obesity may demonstrate different clinical outcomes as compared to their counterparts without obesity. Research suggests that HF patients with obesity have lower rates of hospitalization and mortality than do those with normal or low weights (e.g. Sharma et al., 2015), typically described as the “obesity paradox” (Clark et al., 2014; Lavie et al., 2016), though this may be in contention (Eckel et al., 2018; Khan et al., 2018). There are a number of neural, biological, and behavioral reasons why patients with obesity do not show expected clinical outcomes. Patients with obesity tend to be younger and demonstrate symptoms earlier (Clark et al., 2014; Lavie et al., 2016); it is possible that anxiety in these patients is attributable to factors outside of cognitive function, such as symptom severity.

### Limitations

There are several limitations that should be addressed. While the assessed sample size was sufficiently powered for our statistical models, it was too small to adequately power interaction effects and as such these results should be interpreted with caution. Our sample was at high risk for readmission, and as such we do not know whether these results would generalize to the wider HF population. Unfortunately, due to limits on patient record access or inconsistent charting procedures, we were unable to assess variables such as socioeconomic status, antidepressant use, natriuretic peptide levels, or functional class, that may have impacted the relationships described herein. We do not know the cognitive function of these patients prior to hospitalization, and thus cannot control for this variable. In addition, the timing of assessment during the course of patient’s hospitalization was not controlled, thus patients had variable lengths of stay prior to and following assessment administration that we were not able to control for. Despite limited control of variables when assessing patients while hospitalized, gathering information during hospitalization is critical, as factors that lead to readmission may not otherwise become apparent until the incidence of that readmission, and it may offer an opportunity to intervene clinically before the patient is discharged. Lastly, we did not have access to a control sample of patients without HF, or with a different form of cardiovascular disease. Future research would benefit from between-group comparisons to determine if these relationships are unique to HF, or if this phenomenon is transdiagnostic.

## Conclusion and Clinical Implications

Cognitive dysfunction and psychological distress have been shown to be closely related in the general population, with a growing literature demonstrating this in HF (Foster et al., 2011; Garcia et al., 2011; Alosco et al., 2014a; Hawkins et al., 2015a). Interestingly, treatment of depression may support cognitive function. Antidepressants may help to slightly improve cognitive function (Gallassi et al., 2006), and behavioral activation has been shown to not only improve depressive symptoms, but also help prevent cognitive and functional decline in a community sample (Rovner et al., 2018). Additional inquiry is necessary to determine if treatments for depression can improve cognition in HF, or if treatment outcomes differ by obesity status. Unfortunately, it less well-known how anxiety impacts cognitive function in HF, and whether treatments for anxiety similarly would help to improve cognitive function. This study is the first we are aware of linking anxiety to cognitive function in HF, particularly in patients without obesity. Research suggests that cognitive function can improve in the HF population (Stanek et al., 2009, 2011; Alosco et al., 2014a), and that self-care can improve despite cognitive dysfunction (Cameron et al., 2017). Given the relationship with morbidity, hospitalization, and mortality in HF (Yohannes et al., 2010; Sherwood et al., 2011; Kato et al., 2012; Ketterer et al., 2014; Agarwal et al., 2016; Huynh et al., 2016; Tovar et al., 2016), cognitive assessment in HF is of critical importance, particularly in those patients at high risk for readmission. This research highlights cognitive dysfunction, depression, and anxiety as critical targets in HF treatment, and further emphasizes the need to identify populations at elevated risk.

## Data Availability Statement

The datasets generated for this study are available on request to the corresponding author.

## Ethics Statement

This study was determined to be exempt from 45 CFR 46 Research Regulations by the Institutional Review Board of Christiana Care. A Waiver of HIPAA Authorization was granted, given the study involved no more than minimal risk to the privacy of individuals and as such written informed consent for participation was not required for this study in accordance with the national legislation and the institutional requirements.

## Author Contributions

AE, CA, MB, and EW conducted assessments with patients. AE conceived of the research question and analyzed the data. DB assisted with chart review and analysis. AE, CA, DB, and EW were involved in writing the manuscript and had final approval of the submitted and published versions.

## Conflict of Interest

The authors declare that the research was conducted in the absence of any commercial or financial relationships that could be construed as a potential conflict of interest.
