# Supplementary material for: Relationship Between Psychological Distress and Cognitive Function Differs as a Function of Obesity Status in Inpatient Heart Failure
Source: Front Psychol. 2020 Feb 14;11:162. doi: 10.3389/fpsyg.2020.00162 (PMC7033423; doi:10.3389/fpsyg.2020.00162)
Supplement: Supplementary file 1 [file Data_Sheet_1.pdf]

SUPPLEMENTAL MATERIAL

S1. METHODS

S1.1. Readmission Risk Prediction Model

Health Catalyst's *catalyst.ai* model-driven machine learning and analytics technology was used to train a prediction model using data collected from Christiana Hospitals electronic medical records. *Catalyst.ai* is built using *healthcare.ai* open-source solution designed to streamline healthcare machine learning. Logistic Regression & Random Forrest algorithms were used to build these models. The model was trained using 18 months of patients' data (admitted 7/1/2016 through 12/31/2017) and validated against patients admitted 1/1/2018 through 3/31/2018. This validation included a comparison against the statistics on true positives, true negatives, false positives and false negatives at each risk level. Sensitivity and Specificity stats were reviewed for each level to assess the true positive rate and the true negative rate. After reviewing these stats, a threshold was selected to balance the risk of missing a readmission patient vs. wasting resources on too many patients. The model was then implemented to calculate prediction scores for all admitting patients starting 2/1/2018. These scores were evaluated against actual outcomes until it was deployed to production and embedded within the workflow in July 2018.

S1.1.1. List of Variables included in prediction model

- Admission Type
- Admission Source
- Gender
- Age
- Marital Status
- Lab Results (collected within 36 hrs of admission and most recent)
    - Glucose
    - Sodium
    - WBC
    - Hemoglobin
    - BNP
    - BUN
    - Creatinine
    - Bilirubin
- Vital Sign (collected within 36 hrs of admission and most recent)
    - Heart Rate
    - BP – Systolic and Diastolic
    - Respiratory Rate
    - Pulse OX
    - Temperature
    - Blood Pressure Mean
    - Pulse Pressure
- Ejection Fraction
- History Of Excess Days In Acute Care
- History Of Excess Visits In Acute Care
- Past Year Inpatient Visit Count
- Prior Six Months Emergency Visit Count

Prior Readmit Count
Prior Inpatient Average Length of Stay
Prior Inpatient Sum Length of Stay
Prior Year HF Visit Count
Prior Six Months HF Visit Count
Comorbidity
- Afib
- Cardiomyopathy Flag
- Dementia Flag
- Diabetes Flag
- Psychoses Flag
- Stroke TIA Flag
- Discharge Medication Count

Potential Drug Abuse
- Homeless
- Family Support

Guideline-Directed Medical Therapy administered (within 36 hrs of admission)
- Beta Blocker
- ACEARB
- Aldosterone Antagonist
- Hydralazine Nitrate
- Anticoagulant

Diuretics administered(within 36 hrs of admission)
- FUROSemide Y/N
- FUROSemide Total Dose
- FUROSemide Latest Dose
- Bumetanide Y/N
- Bumetanide Total Dose
- Bumetanide Latest Dose

Intubation
- Intubation Y/N

Length of Stay

S1.2. Inclusion criteria for referral to Heart Failure Task Force
Patients were referred to the HF Task Force to be assessed if they were 1) 18 years of age and older, 2) admitted to the hospital in units within the Cardiovascular and Acute Medicine service lines and 3) have risk score of 0.195 or higher as determined by the *catalyst.ai* model for diagnosis with HF.

S1.3. Exclusion criteria for referral to Heart Failure Task Force
Patients were excluded from HF Task Force assessment 1) if barriers existed to outpatient follow-up (e.g. lived out of state, institutionalized), 2) they were currently followed by a separate cardiology practice, 3) other medical comorbidities that required primary focus during their hospitalization (e.g. end-stage renal disease, severe lung disease). On occasion patients' primary cardiologists would refuse referral for HF Task Force assessment.
